# Effects of curcumin on colonic anastomotic healing in rats: an experimental study

**DOI:** 10.1590/acb416326

**Published:** 2026-11-09

**Authors:** Mehmet Ali Yağcı, Yavuz Atakan Sezer

**Affiliations:** 1 Anka Hospital – Department of Gastrointestinal Surgery – Gaziantep – Turkey.; 2 Trakya University – Faculty of Medicine – Department of General Surgery – Edirne – Turkey.

**Keywords:** Curcumin, Anastomosis, Surgical, Colon, Wound Healing, Oxidative Stress, Rats, Wistar

## Abstract

**Purpose:**

To investigate the effects of curcumin administration on colonic anastomotic healing in rats using mechanical, biochemical, oxidative stress, and histopathological parameters.

**Methods:**

Twenty-eight adult male Wistar Albino rats which underwent distal colonic resection and end-to-end colocolic anastomosis were allocated into four groups: postoperative day 3 (POD3) control, POD3 curcumin, postoperative day 7 (POD7) control, and POD7 curcumin. Curcumin was administered by orogastric gavage at 200 mg/kg/day for seven days before surgery and continued until the predefined assessment day. Anastomotic bursting pressure was the primary outcome.

**Results:**

Curcumin-treated animals had higher bursting pressure than controls overall (186.14 ± 81.13 *versus* 113.57 ± 60.28 mmHg; *p* < 0.001), with significant postoperative day and treatment × day interaction effects (both *p* < 0.001). Hydroxyproline concentration did not show a significant treatment effect (10.52 ± 5.54 *versus* 9.15 ± 3.54 mg/g tissue; *p* = 0.212). Plasma malondialdehyde was lower in curcumin-treated animals (3.83 ± 0.96 *versus* 5.24 ± 1.28 nmol/mL; *p* = 0.004), whereas blood reduced glutathione did not differ significantly (*p* = 0.954).

**Conclusion:**

Curcumin improved anastomotic biomechanical stability and reduced lipid peroxidation, suggesting that its effect on early colonic anastomotic healing may be partly related to oxidative stress modulation.

## Introduction

Colonic anastomosis is a central step in colorectal surgery and is performed to restore intestinal continuity after resection for malignant, inflammatory, obstructive, ischemic, or traumatic diseases. Although contemporary colorectal surgery has benefited from better stapling devices, minimally invasive platforms, enhanced recovery protocols, refined perioperative care, and improved understanding of bowel perfusion, the basic surgical requirement remains unchanged: the anastomosis must heal securely under continuous mechanical, bacterial, vascular, and inflammatory stress^1,2^.

Anastomotic leakage is therefore still one of the most feared complications after colorectal surgery. Its reported incidence varies widely, mainly because of differences in anastomotic level, patient risk profile, operative conditions, and definitions used in the literature^2-4^. Beyond being a local technical failure, leakage may lead to intra-abdominal sepsis, reoperation, prolonged hospitalization, stoma formation, increased costs, impaired quality of life, and higher mortality^2,3^. In oncological surgery, it has also been associated with adverse long-term outcomes, including local recurrence and reduced survival^3^. For these reasons, safe anastomotic healing remains a persistent clinical and experimental priority.

The success of a colonic anastomosis depends on several local and systemic factors. Adequate tissue perfusion, absence of excessive tension, gentle tissue handling, secure mechanical construction, and control of contamination are key local determinants, whereas age, comorbidity, malnutrition, anemia, diabetes, steroid use, smoking, radiotherapy, infection, and emergency conditions may impair healing at the systemic level^3-5^. These factors often converge on a common biological pathway involving inflammation, tissue oxygenation, fibroblast activity, collagen synthesis and degradation, angiogenesis, and extracellular matrix remodeling^6^. Thus, anastomotic failure should be understood not only as a surgical event, but also as the result of disturbed tissue repair.

Experimental models remain important for clarifying these mechanisms and for testing interventions that may improve anastomotic healing. Rat models are commonly used because they are reproducible, technically feasible, and suitable for mechanical, biochemical, and histopathological assessment^7,8^. Anastomotic bursting pressure is frequently used to evaluate early mechanical strength, whereas tissue hydroxyproline concentration reflects collagen content and provides a biochemical estimate of matrix deposition^9-11^. Histopathological evaluation adds complementary information regarding inflammatory infiltration, fibroblast proliferation, neovascularization, collagen organization, and mucosal repair^8,11^. Together, these outcomes allow a broader assessment of anastomotic integrity than any single parameter alone.

Oxidative stress is another relevant component of intestinal wound repair. Surgical trauma, local inflammation, bacterial contamination, and ischemia-reperfusion injury may increase reactive oxygen species, which can damage cellular membranes, alter protein structure, impair fibroblast function, and interfere with collagen metabolism^12^. Malondialdehyde (MDA) is commonly used as a marker of lipid peroxidation, whereas glutathione (GSH) represents an important endogenous antioxidant defense mechanism^12-14^. Previous experimental studies on colonic anastomoses have shown that interventions affecting oxidative and nitro-oxidative stress may influence bursting pressure, hydroxyproline levels, and tissue healing parameters^13,14^. Therefore, evaluating oxidant-antioxidant balance may help explain the biological environment in which anastomotic healing occurs.

Curcumin, the principal polyphenolic compound derived from *Curcuma longa*, has attracted interest because of its antioxidant, anti-inflammatory, antimicrobial, and wound-healing properties^15^. Experimental wound-healing studies, including diabetic and impaired-healing models, have shown that curcumin may accelerate wound closure, enhance re-epithelialization, promote fibroblast migration and neovascularization, support collagen deposition, modulate transforming growth factor-β-related pathways, reduce inflammatory cytokine expression, improve antioxidant enzyme activity, and support granulation tissue formation^16–19^. In diabetic and impaired wound-healing models, curcumin has also been associated with reduced inflammatory cytokine expression, improved antioxidant enzyme activity, and better granulation tissue formation17,18. More recently, oral curcumin supplementation has been evaluated in abdominal surgical wound healing in rats and was associated with reduced inflammatory parameters^20^.

Despite these biological properties, the effect of curcumin on colonic anastomotic healing remains insufficiently defined when mechanical strength, collagen-related biochemical repair, systemic oxidative stress markers, and histopathological findings are evaluated together. The present experimental study aimed to investigate the effects of curcumin administration on colonic anastomotic healing in rats by assessing anastomotic bursting pressure, perianastomotic tissue hydroxyproline concentration, plasma MDA, blood GSH levels, and histopathological findings on postoperative days 3 and 7.

## Methods

### Study design

This was a controlled experimental animal study designed to evaluate the effect of curcumin on colonic anastomotic healing in rats. The study was conducted at the Experimental Animals Research Unit of Trakya University Faculty of Medicine after approval by the Local Ethics Committee of Trakya University Faculty of Medicine (TÜHDYEK-2009/003; decision no. 4, March 30, 2009). The manuscript was prepared in accordance with the ARRIVE 2.0 recommendations for reporting animal research^21^.

The experimental design consisted of four independent groups defined by treatment condition and postoperative assessment day. The experimental unit was the individual rat. Postoperative day 3 and postoperative day 7 assessments were derived from predefined group allocation and did not represent repeated measurements within the same animal.

### Animals and housing

Twenty-eight adult male Wistar Albino rats were used. The mean body weight was 210 g, with a range of 180–230 g. The animals were housed under controlled laboratory conditions at 22 ± 1°C, with a 12-hour light/dark cycle and 50–60% humidity. Standard pellet chow and tap water were provided until the experimental procedure. The animals were fasted for 12 hours before surgery, but access to water was maintained. All the procedures were done in Trakya University Faculty of Medicine.

### Experimental groups and curcumin administration

The rats were divided into four groups, with seven animals in each group. All animals underwent distal colonic resection and end-to-end colocolic anastomosis. In the present study, the term “vehicle control” was used to denote the non-curcumin control animals that underwent the same surgical procedure and were evaluated at the same postoperative time points as the curcumin-treated animals. To ensure identical handling and procedure-related stress levels across all experimental arms, control group animals received an equal volume of 0.9% sterile saline solution via orogastric gavage daily, mirroring the administration protocol of the treatment groups.

- Group 1 was defined as the POD3 vehicle control group. These animals underwent colonic anastomosis and were evaluated on postoperative day 3.
- Group 2 was defined as the POD3 curcumin group. These animals received curcumin at a dose of 200 mg/kg/day by orogastric gavage for seven days before surgery. Curcumin administration continued after surgery until evaluation on postoperative day 3.
- Group 3 was defined as the POD7 vehicle control group. These animals underwent colonic anastomosis and were evaluated on postoperative day 7.
- Group 4 was defined as the POD7 curcumin group. These animals received curcumin at a dose of 200 mg/kg/day by orogastric gavage for seven days before surgery. Curcumin administration was continued after surgery until evaluation on postoperative day 7.

### Anesthesia and surgical procedure

No mechanical bowel preparation was performed before surgery. Anesthesia was induced by intramuscular administration of xylazine hydrochloride at 5–10 mg/kg and ketamine hydrochloride at 50 mg/kg. After anesthesia, the animals were placed in the supine position and fixed to the operating table. The abdominal hair was shaved, and the surgical field was prepared with povidone-iodine solution.

A midline laparotomy was performed. The descending colon was identified, and a 1-cm segment of colon was resected 3 cm proximal to the peritoneal reflection. End-to-end colocolic anastomosis was then performed using 6-0 polypropylene sutures in a single-layer, interrupted, inverting fashion. Six sutures were placed for each anastomosis. All anastomoses were performed by the same surgeon to reduce technical variability. After completion of the anastomosis, the abdominal wall was closed with interrupted 3-0 silk sutures.

### Postoperative assessment and sample collection

Animals in the POD3 vehicle control and POD3 curcumin groups were evaluated on postoperative day 3, whereas animals in the POD7 vehicle control and POD7 curcumin groups were evaluated on postoperative day 7. At relaparotomy, the previous incision line was reopened, and the anastomotic region was identified. The perianastomotic area was inspected macroscopically. The colon was then transected 3 cm proximal and 3 cm distal to the anastomotic line, and a 6-cm colonic segment containing the anastomosis at its center was removed for mechanical testing. Perianastomotic tissue samples were obtained for biochemical and histopathological analyses.

The primary outcome was anastomotic bursting pressure. Secondary outcomes included perianastomotic tissue hydroxyproline concentration, plasma MDA, blood reduced GSH, MDA/GSH ratio, and histopathological healing scores.

### Anastomotic bursting pressure

#### System standardization

Before mechanical testing, the pressure-measuring system was standardized for all specimens. The digital/aneroid sphygmomanometer used for intraluminal pressure measurement was calibrated immediately before the experimental procedures. The same catheter, ligature material, water-filled container, and pressure-measurement setup were used throughout the experiment. The resected colonic segment was carefully prepared without disrupting the anastomotic line. A catheter was inserted into the bowel lumen, and both ends of the segment were securely ligated with 3-0 silk sutures to prevent leakage from the bowel ends or catheter insertion site.

#### Pacing and precision

Methylene blue was injected into the bowel lumen to facilitate visual identification of leakage. The prepared colonic segment was then submerged in a water-filled container and connected to the calibrated pressure-measurement system. Inflation was performed using an automated/controlled infusion assembly at a continuous low flow rate of approximately 2–4 mmHg/sec. This standardized pacing was used to avoid abrupt pressure spikes and to improve the reproducibility of bursting pressure measurements.

#### Objective criterion

Anastomotic bursting pressure was defined as the exact intraluminal pressure, measured in mmHg, at which the first visual leak from the anastomotic line was observed. Leakage was identified by the appearance of air bubbles and methylene blue extravasation from the anastomotic line. If a sudden pressure drop was recorded at the same point, this was also accepted as confirming anastomotic failure. Leakage from the bowel ends or catheter entry site was not accepted as an anastomotic burst. The recorded pressure value was used as the anastomotic bursting pressure for that specimen.

### Perianastomotic tissue hydroxyproline analysis

Perianastomotic tissue samples were dried for 24 hours and then hydrolyzed in hydrochloric acid for 24 hours. The liquid phase of the hydrolysate was evaporated under nitrogen gas. Hydroxyproline residues in the prepared hydrolysate were measured spectrophotometrically after reaction with chloramine-T, using a modified colorimetric method for hydroxyproline analysis^22^.

Standard solutions of L-hydroxyproline at concentrations of 10, 25, and 50 mg/dL were prepared. The standard curve equation was calculated by regression analysis as y = -0.006 + 0.02x. Hydroxyproline concentrations in the tissue samples were calculated using this equation and normalized to dry tissue weight. Results were expressed as mg/g tissue.

### Plasma malondialdehyde analysis

Plasma MDA levels were measured as an indicator of lipid peroxidation. The analysis was based on the spectrophotometric measurement of the pink-red chromogen formed by the reaction between MDA and thiobarbituric acid under heated conditions^23^. Absorbance was measured at 532 nm. All measurements were performed twice. MDA concentrations were calculated using the molar absorptivity of MDA at 532 nm and expressed as nmol/mL.

### Blood reduced glutathione analysis

Blood GSH levels were measured using a colorimetric method based on the reaction of sulfhydryl groups with 5,5’-dithiobis (2-nitrobenzoic acid) acid under alkaline conditions^24^. The yellow-colored product formed during this reaction was measured spectrophotometrically at 412 nm. The intensity of absorbance was proportional to the GSH concentration. Results were expressed as µmol/L/dL red blood cells (RBC).

### Histopathological evaluation

Perianastomotic tissue specimens were evaluated by the pathology team for inflammatory cell infiltration, neovascularization, and fibroblastic activity. The histopathological scoring system included four ordinal parameters: polymorphonuclear leukocyte (PNL) infiltration, mononuclear cell (MNC) infiltration, blood vessel neodevelopment, and fibroblastic activity.

PNL infiltration was scored as 0 for absence, 1 for sparse PNL infiltration, 2 for small PNL clusters, and 3 for large PNL clusters or diffuse infiltration. MNC infiltration was scored as 0 for absence, 1 for sparse MNC infiltration, 2 for focal MNC accumulation, and 3 for diffuse infiltration. Blood vessel neodevelopment was scored as 0 for absence, 1 for 1–10 vessels, 2 for 11–20 vessels, and 3 for more than 20 vessels per high-power field. Fibroblastic activity was scored as 0 for absence, 1 for sparse fibroblasts, 2 for focal fibrosis, and 3 for diffuse fibrosis.

### Statistical analysis

Statistical analyses were performed using Python with pandas, numpy, scipy, and statsmodels libraries. The experimental design consisted of four independent groups defined by treatment condition and postoperative assessment day: POD3 vehicle control, POD3 curcumin, POD7 vehicle control, and POD7 curcumin. Accordingly, the original four-group structure was recoded into two factorial variables: treatment condition and postoperative assessment day. Treatment condition was coded as vehicle control or curcumin, while postoperative assessment day was coded as POD3 or POD7. The postoperative assessment day was derived from the predefined experimental group allocation and did not represent repeated measurements within the same animal.

Continuous variables were summarized as mean ± standard deviation. Ordinal histopathological scores were summarized as median with minimum–maximum values. The primary outcome was anastomotic bursting pressure. Secondary continuous outcomes included hydroxyproline concentration, plasma MDA, blood GSH, and MDA/GSH ratio. Histopathological outcomes included PNL infiltration, MNC infiltration, blood vessel neodevelopment, and fibroblastic activity scores.

For continuous outcomes, the primary analysis was performed using a 2 × 2 factorial linear model including treatment condition, postoperative assessment day, and the treatment × postoperative assessment day interaction. This model was used to evaluate three prespecified effects: the overall treatment effect of curcumin versus vehicle control, the overall effect of postoperative assessment day, and whether the treatment effect differed between POD3 and POD7. Model assumptions were evaluated using the Shapiro-Wilk’s test for residual normality and the Brown-Forsythe/Levene’s test for homogeneity of variances. When model assumptions were adequately met, standard Type-II factorial analysis of variance (ANOVA) was used. When variance homogeneity was violated, HC3 robust Type-II factorial ANOVA was applied.

For interpretation of the factorial model, marginal summaries were calculated for treatment condition and postoperative assessment day. The treatment main effect was presented by comparing all curcumin-treated animals with all vehicle control animals. The postoperative assessment day main effect was presented by comparing all POD7 animals with all POD3 animals. The interaction effect was interpreted as the difference between the curcumin-control difference at POD7 and the curcumin-control difference at POD3.

In addition to the primary factorial analysis, four-group omnibus comparisons were performed as supplementary analyses to allow comparability with the conventional group-based approach. For continuous outcomes with acceptable variance homogeneity, one-way ANOVA was used followed by Tukey’s honestly significant difference post hoc test when the omnibus test was significant. For outcomes with unequal variances, Welch ANOVA was used followed by pairwise Welch tests with Holm adjustment. These four-group analyses were considered supportive and were not used as the primary basis for inference.

Histopathological scores were analyzed as ordinal outcomes. Therefore, cumulative logit ordinal regression models were fitted using treatment condition, postoperative assessment day, and their interaction as predictors. For supplementary four-group comparisons of histopathological scores, Kruskal-Wallis’ tests were used. When appropriate, post hoc pairwise comparisons were performed using Dunn’s tests with Holm adjustment for multiple comparisons. Median shifts were reported descriptively for ordinal outcomes, but inferential interpretation was based on the ordinal regression models and non-parametric post hoc tests.

The association between hydroxyproline concentration and anastomotic bursting pressure was evaluated using Spearman’s correlation analysis. In addition, an adjusted partial Spearman’s correlation analysis was performed to account for treatment condition and postoperative assessment day. For the adjusted analysis, hydroxyproline concentration and bursting pressure were first rank transformed. The rank-transformed values were then residualized against treatment condition and postoperative assessment day using linear regression models. Spearman’s correlation was subsequently calculated between the resulting residuals. This approach was used to determine whether the relationship between hydroxyproline concentration and bursting pressure persisted after adjustment for the predefined experimental group structure.

All tests were two-sided. A p-value < 0.05 was considered statistically significant. For post hoc analyses, adjusted p-values were interpreted. No imputation was performed because there were no missing data. The results were reported with exact *p*-values when possible, and p-values < 0.001 were reported as < 0.001.

## Results

### Mechanical strength of the anastomosis

Continuous mechanical, biochemical, and oxidative stress parameters are presented in Table 1. Anastomotic bursting pressure was the primary outcome and showed the clearest treatment-related difference. Bursting pressure was higher in the curcumin-treated groups than in the vehicle control groups on both postoperative day 3 (POD3) and postoperative day 7 (POD7). On POD3, mean bursting pressure increased from 56.00 ± 8.25 mmHg in vehicle controls to 109.14 ± 9.23 mmHg in the curcumin group. On POD7, the corresponding values were 171.14 ± 8.40 mmHg and 263.14 ± 18.47 mmHg, respectively.

**Table 1:** Mechanical, biochemical, and oxidative stress parameters according to treatment condition and postoperative assessment day.

		Biochemical mechanical and oxidative stress parameters								
		Bursting pressure (mmHg)		Hydroxyproline (mg/g tissue)		Plasma MDA (nmol/mL)		Blood GSH (µmol/L/dL RBC)		MDA/GSH ratio
		mean ± SD		mean ± SD		mean ± SD		mean ± SD		mean ± SD
**POD3 vehicle control (n = 7)**	**: G1**	56.00 ± 8.25		11.64 ± 3.12		5.28 ± 0.97		25.03 ± 3.18		0.21 ± 0.04
**POD3 curcumin (n = 7)**	**: G2**	109.14 ± 9.23		13.19 ± 6.85		4.02 ± 0.90		26.49 ± 3.71		0.15 ± 0.04
**POD7 vehicle control (n = 7)**	**: G3**	171.14 ± 8.40		6.66 ± 1.74		5.19 ± 1.62		23.66 ± 1.70		0.22 ± 0.07
**POD7 curcumin (n = 7)**	**: G4**	263.14 ± 18.47		7.85 ± 1.74		3.64 ± 1.04		22.34 ± 3.69		0.17 ± 0.05
**Total (n = 28)**	**: G5**	149.86 ± 79.27		9.84 ± 4.62		4.53 ± 1.32		24.38 ± 3.38		0.19 ± 0.06
**Vehicle control (n = 14)**	**: G1 + G3**	113.57 ± 60.28		9.15 ± 3.54		5.24 ± 1.28		24.34 ± 2.55		0.22 ± 0.05
**Curcumin (n = 14)**	**: G2 + G4**	186.14 ± 81.13		10.52 ± 5.54		3.83 ± 0.96		24.41 ± 4.15		0.16 ± 0.04
**Δ (Curcumin−control)**	**: (G2 + G4) - (G1 + G3)**	72.57		1.37		-1.41		0.07		-0.06
**Treatment *p* -value**		< 0.001^a^		0.212^b^		0.004^a^		0.954^a^		0.007^a^
**POD3 (G1 + G2, n = 14)**	**: G1 + G2**	82.57 ± 28.83		12.42 ± 5.18		4.65 ± 1.11		25.76 ± 3.41		0.18 ± 0.05
**POD7 (G3 + G4, n = 14)**	**: G3 + G4**	217.14 ± 49.69		7.26 ± 1.78		4.42 ± 1.54		23.00 ± 2.84		0.19 ± 0.06
**Δ (POD7−POD3)**	**: (G3 + G4) - (G1 + G2)**	134.57		-5.16		-0.23		-2.76		0.01
**Assessment day *p* -value**		< 0.001^a^		< 0.001^b^		0.603^a^		0.031^a^		0.625^a^
**Δ POD3 (Curcumin−Control)**	**: G2-G1**	53.14		1.55		-1.26		1.46		-0.06
**Δ POD7 (Curcumin−Control)**	**: G4-G3**	92		1.19		-1.55		-1.32		-0.05
**ΔΔ shift**	**: (G4-G3) - (G2-G1)**	38.86		-0.36		-0.29		-2.78		0
**Interaction *p* -value**		< 0.001^a^		0.913^b^		0.744^a^		0.259^a^		0.911^a^
**Omnibus *p* -value**		< 0.001^e^		0.013^f^		0.030^e^		0.114^e^		0.052^e^
**Pairwise comparison *p* -values**										
**G1 *versus* G2**		< 0.001^h^		0.601^i^		0.208^h^		0.825^h^		0.164^h^
**G1 *versus* G3**		< 0.001^h^		0.028^i^		0.999^h^		0.851^h^		0.993^h^
**G1 *versus* G4**		< 0.001^h^		0.099^i^		0.066^h^		0.407^h^		0.332^h^
**G2 *versus* G3**		< 0.001^h^		0.183^i^		0.262^h^		0.363^h^		0.099^h^
**G2 *versus* G4**		< 0.001^h^		0.262^i^		0.929^h^		0.096^h^		0.973^h^
**G3 *versus* G4**		< 0.001^h^		0.451^i^		0.087^h^		0.864^h^		0.217^h^

a Standard Type-II 2×2 factorial analysis of variance (ANOVA);

b HC3 robust Type-II 2×2 factorial ANOVA;

e one-way ANOVA;

f Welch one-way ANOVA pairwise cells show multiplicity-adjusted post hoc p-values;

h Tukey’s honestly significant difference post hoc test;

i pairwise Welch t test with Holm adjustment;

SD: standard deviation; POD: postoperative day; G1: POD3 vehicle control; G2: POD3 curcumin; G3: POD7 vehicle control; G4: POD7 curcumin; MDA: malondialdehyde; GSH: glutathione; RBC: red blood cells. Source: Elaborated by the authors.

In the 2 × 2 factorial analysis, curcumin administration had a significant treatment effect on bursting pressure (*p* < 0.001). The postoperative assessment day effect was also significant (*p* < 0.001), indicating higher bursting pressure on POD7 than on POD3. In addition, the treatment × postoperative assessment day interaction was significant (*p* < 0.001), showing that the curcumin-associated increase was greater on POD7 than on POD3. The treatment-related difference was 53.14 mmHg on POD3 and 92.00 mmHg on POD7. Supplementary four-group analysis confirmed a significant difference among groups (*p* < 0.001), and all adjusted pairwise comparisons were significant. These findings are illustrated in Fig. 1.

**Figure 1:**
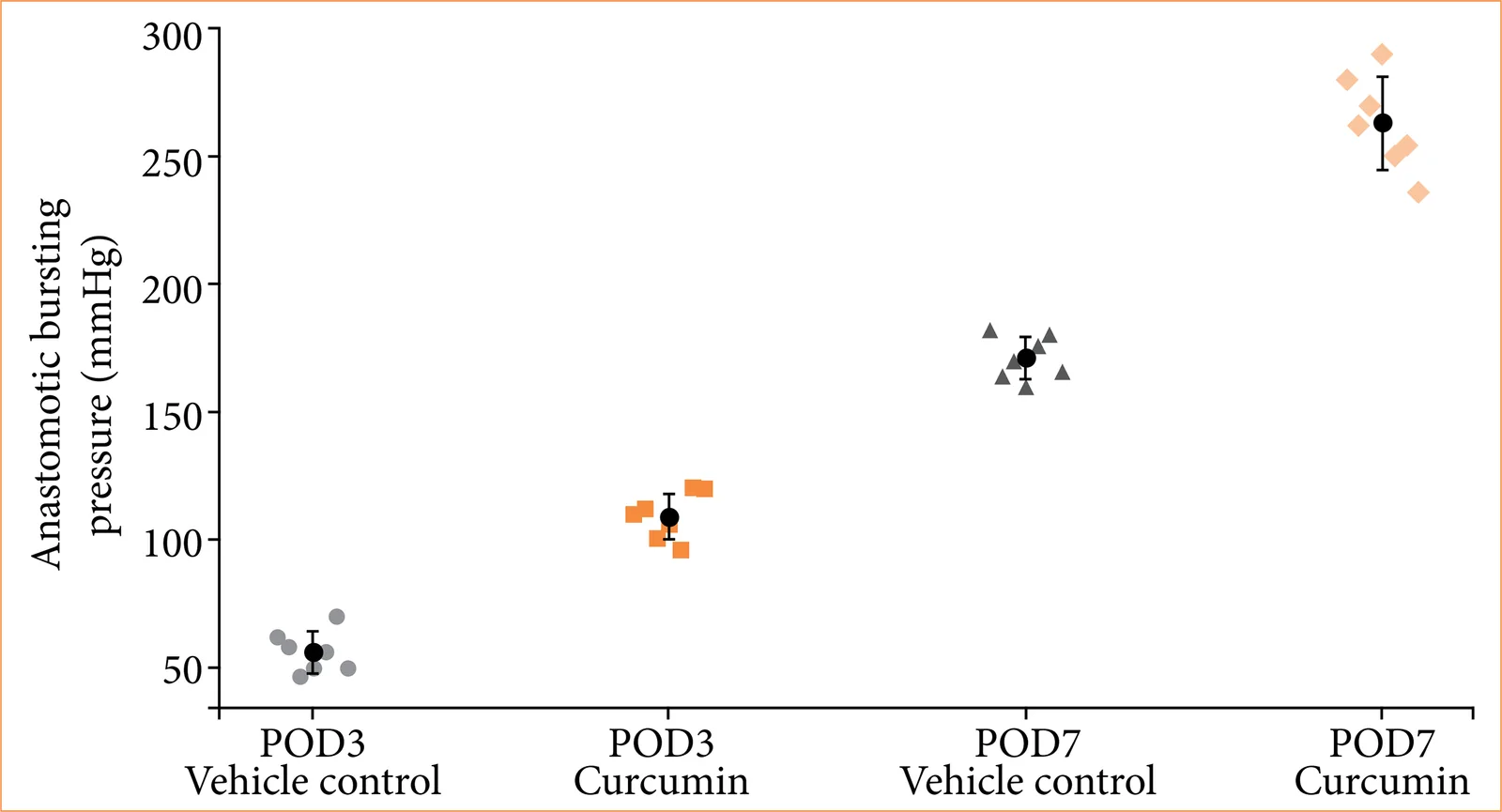
Anastomotic bursting pressure according to treatment condition and postoperative assessment day. Bursting pressure was the primary and strongest outcome of the study. Values were markedly higher in the curcumin groups than in the vehicle control groups at both postoperative day 3 (POD3) and postoperative day 7 (POD7). The treatment effect, postoperative assessment day effect, and treatment × postoperative assessment day interaction were all statistically significant (all *p* < 0.001).

Source: Elaborated by the authors.

### Hydroxyproline concentration

Hydroxyproline concentration did not show a significant overall treatment effect. Across both postoperative assessment days, mean hydroxyproline concentration was 9.15 ± 3.54 mg/g tissue in vehicle controls and 10.52 ± 5.54 mg/g tissue in curcumin-treated animals, corresponding to a mean difference of 1.37 mg/g tissue. This difference was not statistically significant in the factorial model (*p* = 0.212).

The postoperative assessment day effect was significant (*p* < 0.001), with lower hydroxyproline values on POD7 than on POD3. However, the treatment × postoperative assessment day interaction was not significant (*p* = 0.913), indicating that the curcumin-control difference in hydroxyproline did not differ meaningfully between POD3 and POD7. In the supplementary four-group analysis, the overall difference was significant (*p* = 0.013), but adjusted post hoc comparisons showed no significant curcumin-control difference within either POD3 or POD7.

### Oxidative stress parameters

Curcumin-treated animals had lower plasma MDA levels than vehicle controls (Table 1 and Fig. 2). Across both postoperative assessment days, mean plasma MDA was 5.24 ± 1.28 nmol/mL in vehicle controls and 3.83 ± 0.96 nmol/mL in curcumin-treated animals. The factorial model showed a significant treatment effect for plasma MDA (*p* = 0.004). Neither the postoperative assessment day effect (p = 0.603) nor the treatment × postoperative assessment day interaction (*p* = 0.744) was significant.

**Figure 2:**
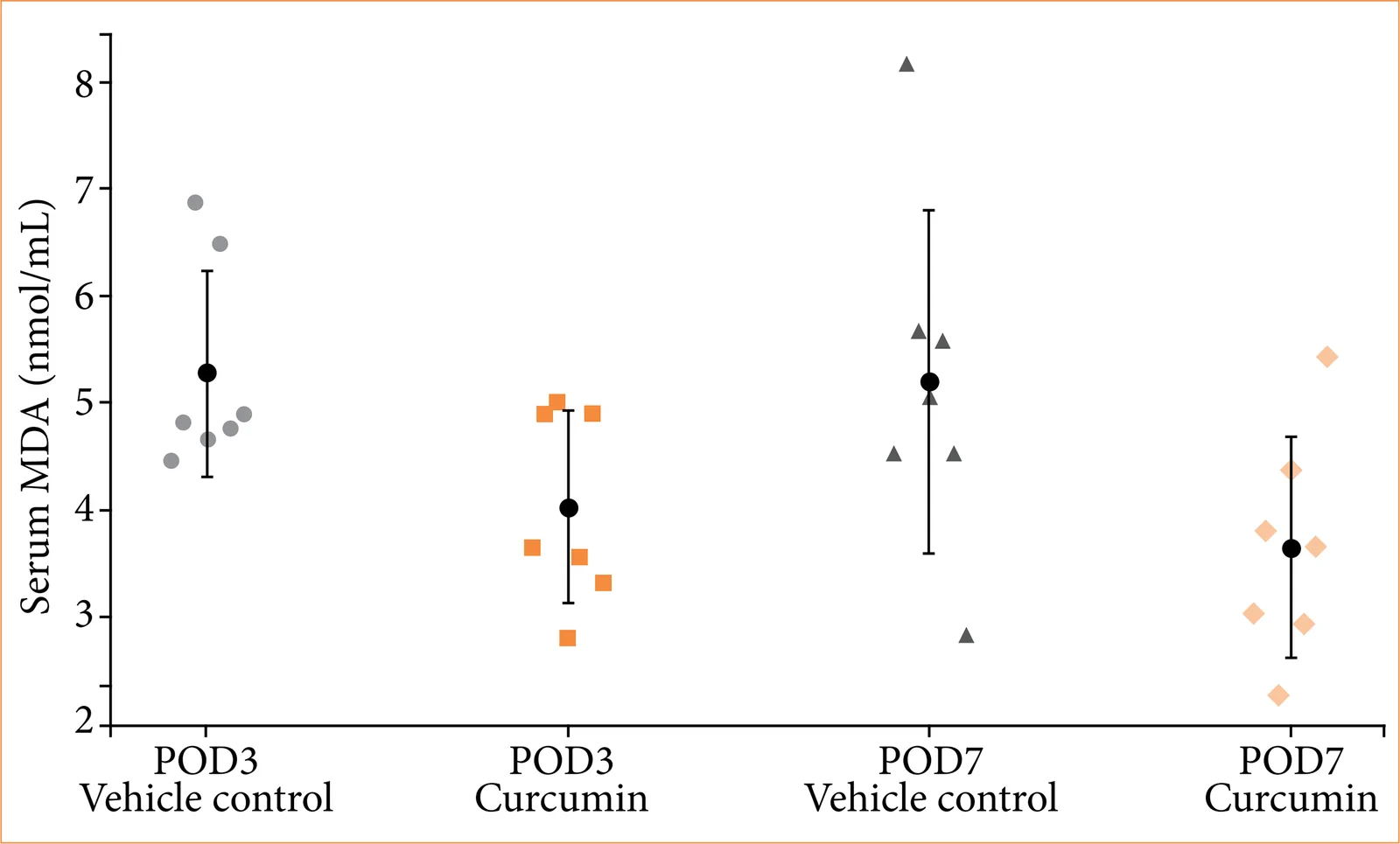
Plasma malondialdehyde (MDA) levels according to treatment condition and postoperative assessment day. Plasma MDA levels were lower in curcumin-treated animals than in vehicle controls. The factorial model showed a significant treatment effect (*p* = 0.004), whereas the postoperative assessment day effect (*p* = 0.603) and the interaction effect (*p* = 0.744) were not significant.

POD3: postoperative day 3; POD7: postoperative day 7. Source: Elaborated by the authors.

Blood GSH levels did not differ significantly according to treatment condition. Mean GSH levels were 24.34 ± 2.55 µmol/L/dL RBC in vehicle controls and 24.41 ± 4.15 µmol/L/dL RBC in curcumin-treated animals, with no significant treatment effect (*p* = 0.954). A significant postoperative assessment day effect was observed (*p* = 0.031), with lower GSH levels on POD7 than on POD3, but the interaction effect was not significant (*p* = 0.259).

The MDA/GSH ratio was evaluated as an exploratory derived index of oxidant-antioxidant balance. The ratio was lower in curcumin-treated animals than in vehicle controls, with a significant treatment effect in the factorial model (*p* = 0.007). No significant postoperative assessment day effect or interaction effect was observed.

### Association between hydroxyproline and bursting pressure

The relationship between hydroxyproline concentration and anastomotic bursting pressure is shown in Fig. 3. In the unadjusted analysis, hydroxyproline concentration showed a significant negative correlation with bursting pressure (Spearman rho = -0.498, *p* = 0.007). However, after adjustment for treatment condition and postoperative assessment day, this association was no longer significant (partial Spearman rho = -0.127, *p* = 0.519). This indicates that the unadjusted association was largely explained by the predefined experimental group structure.

**Figure 3:**
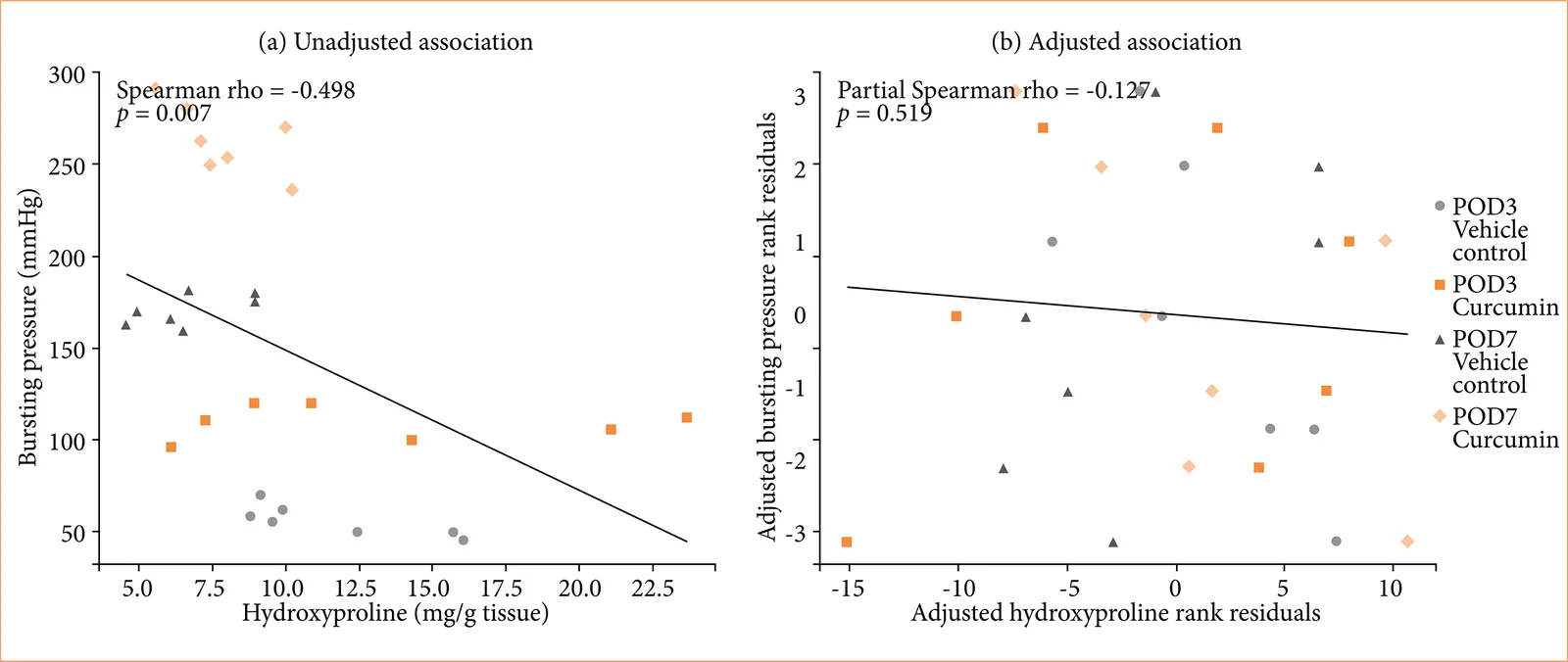
Association between hydroxyproline concentration and anastomotic bursting pressure. (a) Unadjusted association between hydroxyproline concentration and bursting pressure, with a significant negative Spearman’s correlation (rho = -0.498, p = 0.007). (b) Adjusted association after accounting for treatment condition and postoperative assessment day using partial Spearman’s correlation based on rank residuals. After adjustment, the association was no longer statistically significant (partial rho = -0.127, p = 0.519).

Source: Elaborated by the authors.

### Histopathological findings

Histopathological outcomes are presented in Table 2. Polymorphonuclear leukocyte infiltration, mononuclear cell infiltration, blood vessel neodevelopment, and fibroblastic activity were analyzed as ordinal variables and summarized as median values with minimum–maximum ranges.

**Table 2:** Histopathological healing scores according to treatment condition and postoperative assessment day.

		Histopathological outcomes						
		PNL infiltration score		MNC infiltration score		Blood vessel neodevelopment score		Fibroblastic activity score
		Med (min–max)		Med (min–max)		Med (min–max)		Med (min–max)
**POD3 vehicle control (n = 7)**	**: G1**	2 (1–2)		2 (1–2)		1 (1–2)		2 (2–3)
**POD3 curcumin (n = 7)**	**: G2**	1 (1–3)		1 (1–2)		1 (1–2)		1 (1–2)
**POD7 vehicle control (n = 7)**	**: G3**	3 (3–3)		3 (2–3)		3 (1–3)		3 (2–3)
**POD7 curcumin (n = 7)**	**: G4**	3 (1–3)		3 (1–3)		2 (1–2)		2 (1–3)
**Total (n = 28)**	**: G5**	2 (1–3)		2 (1–3)		1 (1–3)		2 (1–3)
**Vehicle control (n = 14)**	**: G1 + G3**	2.5 (1–3)		2 (1–3)		1.5 (1–3)		2 (2–3)
**Curcumin (n = 14)**	**: G2 + G4**	2 (1–3)		2 (1–3)		1 (1–2)		2 (1–3)
**Δ (Curcumin−control)**	**: (G2 + G4) -** **(G1 + G3)**	-0.5		0.0		-0.5		0.0
**Treatment *p* -value**		0.061^k^		0.184^k^		0.172^k^		< 0.001^k^
**POD3 (G1 + G2, n = 14)**	**: G1 + G2**	1 (1–3)		1.5 (1–2)		1 (1–2)		2 (1–3)
**POD7 (G3 + G4, n = 14)**	**: G3 + G4**	3 (1–3)		3 (1–3)		2 (1–3)		2 (1–3)
**Δ (POD7−POD3)**	**: (G3 + G4) -** **(G1 + G2)**	2.0		1.5		1.0		0.0
**Assessment day *p* -value**		< 0.001^k^		< 0.001^k^		0.014^k^		0.004^k^
**Δ POD3 (Curcumin−Control)**	**: G2-G1**	-1.0		-1.0		0.0		-1.0
**Δ POD7 (Curcumin−Control)**	**: G4-G3**	0.0		0.0		-1.0		-1.0
**ΔΔ shift**	**: (G4-G3)** **- (G2-G1)**	1.0		1.0		-1.0		0.0
**Interaction *p* -value**		0.132^k^		0.364^k^		0.211^k^		0.425^k^
**Omnibus *p* -value**		0.001^g^		0.004^g^		0.082^g^		0.004^g^
**Pairwise comparison *p* -values**								
**G1 *versus* G2**		0.821^j^		0.756^j^		0.999^j^		0.081^j^
**G1 *versus* G3**		0.008^j^		0.019^j^		0.152^j^		0.458^j^
**G1 *versus* G4**		0.181^j^		0.316^j^		0.999^j^		0.688^j^
**G2 *versus* G3**		0.004^j^		0.008^j^		0.152^j^		0.002^j^
**G2 *versus* G4**		0.142^j^		0.215^j^		0.999^j^		0.180^j^
**G3 *versus* G4**		0.409^j^		0.405^j^		0.682^j^		0.326^j^

k Cumulative logit ordinal regression likelihood-ratio test;

g Kruskal-Wallis’ test;

j Dunn’s test with Holm adjustment;

POD: postoperative day; G1: POD3 vehicle control; G2: POD3 curcumin; G3: POD7 vehicle control; G4: POD7 curcumin; PNL: polymorphonuclear leukocyte; MNC: mononuclear cell.

Source: Elaborated by the authors.

PNL and MNC infiltration scores were mainly associated with postoperative assessment day rather than treatment condition. Both scores were higher on POD7 than on POD3, with significant postoperative assessment day effects in the ordinal regression models (both *p* < 0.001). Treatment effects were not statistically significant for PNL infiltration (*p* = 0.061) or MNC infiltration (*p* = 0.184), and no significant treatment × postoperative assessment day interactions were detected.

Blood vessel neodevelopment showed a significant postoperative assessment day effect in the ordinal regression model (*p* = 0.014), with higher scores on POD7 than on POD3. However, the treatment effect was not significant (*p* = 0.172), and the supplementary four-group comparison did not reach statistical significance (*p* = 0.082).

Fibroblastic activity showed significant treatment and postoperative assessment day effects in the ordinal regression model (*p* < 0.001 and *p* = 0.004, respectively), while the interaction effect was not significant (*p* = 0.425). However, within-day curcumin-control comparisons did not remain significant after post hoc adjustment. Fibroblastic activity scores are shown in Fig. 4.

**Figure 4:**
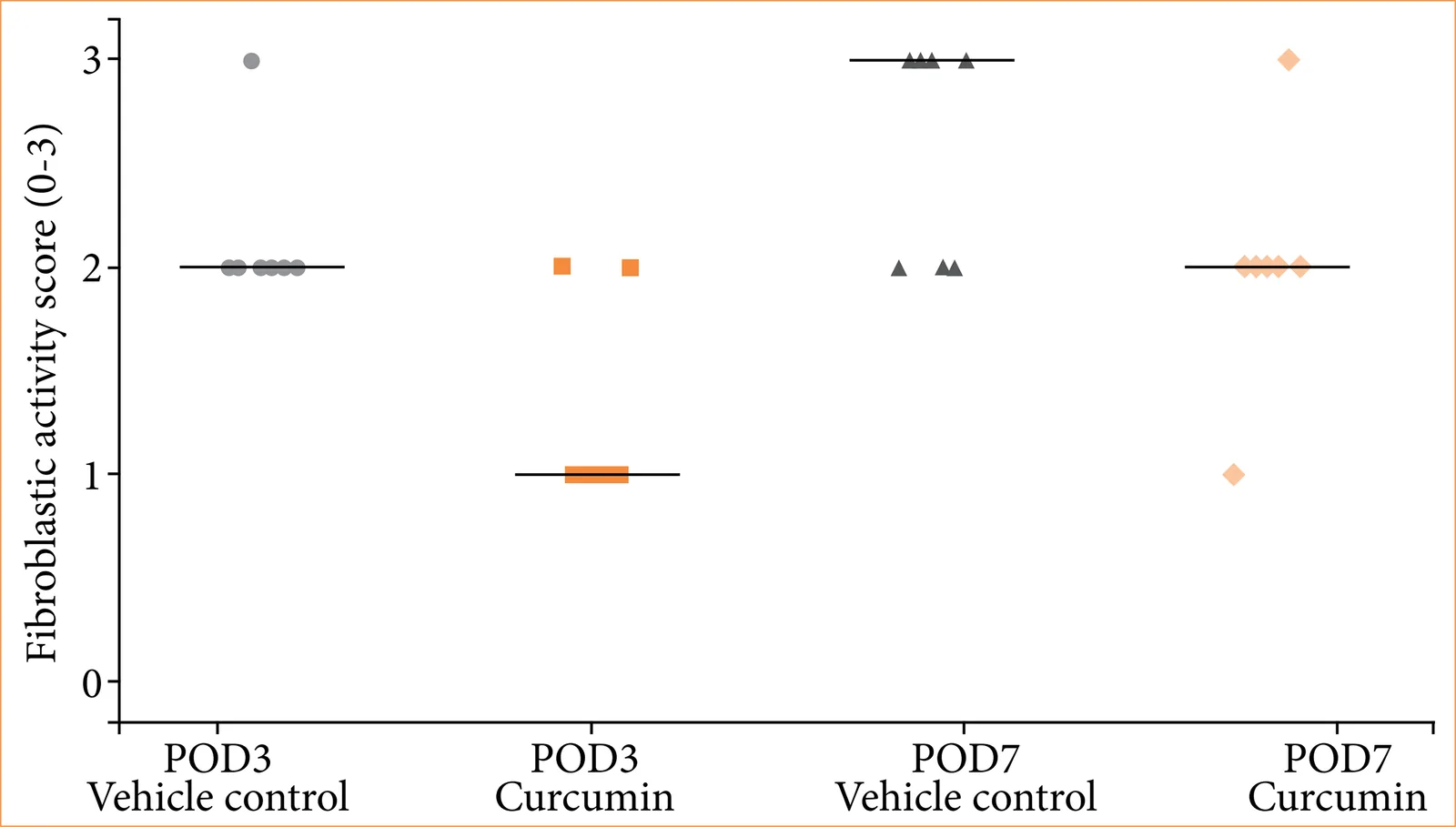
Fibroblastic activity score according to treatment condition and postoperative assessment day. Fibroblastic activity was analyzed as an ordinal outcome. In the ordinal regression model, both the treatment effect (*p* < 0.001) and postoperative assessment day effect (*p* = 0.004) were significant, whereas the interaction effect was not significant (*p* = 0.425).

POD3: postoperative day 3; POD7: postoperative day 7. Source: Elaborated by the authors.

## Discussion

The principal finding of this experimental study was that curcumin administration improved the biomechanical stability of colonic anastomoses during the early healing period. This effect was evident at both postoperative assessment points and became more pronounced by the later phase of evaluation. However, the increase in anastomotic bursting pressure was not accompanied by a parallel or statistically consistent increase in tissue hydroxyproline concentration, suggesting that the mechanical benefit of curcumin cannot be explained solely by an increase in total collagen content. Curcumin was also associated with a reduction in lipid peroxidation, reflected by lower plasma MDA levels, whereas blood GSH levels did not show a treatment-related increase. Therefore, the antioxidant effect observed in this model appears to be driven mainly by reduced oxidative damage rather than by a measurable enhancement of systemic GSH reserve.

Histopathological scores did not show a uniform curcumin-related improvement that fully paralleled the mechanical findings. Most histological changes were more closely related to postoperative healing time than to treatment status, indicating that conventional ordinal histological scoring may not fully capture the biological processes underlying improved anastomotic strength. Overall, these findings suggest that curcumin may enhance early colonic anastomotic integrity through mechanisms that are only partly reflected by standard collagen, oxidative stress, and histopathological parameters.

Poyrazoglu et al.^13^ evaluated the effects of N-acetylcysteine (NAC), ebselen, and 1400W in a rat colonic anastomosis model, focusing on the prevention of oxidative and nitro-oxidative stress. Their study used Sprague-Dawley rats, applied the treatments for four days after anastomosis, and performed relaparotomy on postoperative day 5. The outcomes included anastomotic bursting pressure, tissue MDA, superoxide dismutase (SOD), glutathione peroxidase (GPx), and hydroxyproline. The most favorable profile was observed with 1400W, a selective inducible nitric oxide synthase (iNOS) inhibitor which increased bursting pressure and hydroxyproline while decreasing MDA and SOD levels.

The present study is consistent with this work^13^ in showing that modulation of oxidative injury can be associated with stronger anastomotic repair. However, unlike the 1400W model, curcumin improved bursting pressure without a parallel increase in hydroxyproline. This difference suggests that the biomechanical benefit of curcumin may not be explained primarily by greater total collagen accumulation, but may instead reflect reduced lipid peroxidation, altered inflammatory activity, or improved quality of tissue remodeling.

Canbay et al.^14^ investigated montelukast in Wistar albino rats undergoing colon resection and anastomosis, using both oral and rectal administration routes. Their animals were evaluated on postoperative day 5, and the study assessed anastomotic bursting pressure, hydroxyproline, tissue MDA, GSH, GPx, SOD, and immunohistochemical expression of Ki-67, vascular endothelial growth factor (VEGF), and basic fibroblast growth factor (bFGF). In that model, montelukast reduced MDA and increased antioxidant parameters, but hydroxyproline, Ki-67, and VEGF were decreased, and bursting pressure did not significantly change. Although they evaluated both oral and rectal montelukast administration, the administration route did not significantly modify bursting pressure or hydroxyproline findings, although rectal administration showed a numerically lower bursting pressure and a more pronounced hydroxyproline decrease. This study is particularly useful for interpreting our results because it shows that improvement in oxidative stress markers does not necessarily translate into better mechanical healing. In contrast, curcumin in the present model produced a clear biomechanical benefit despite the absence of a significant hydroxyproline response. The comparison also requires caution because Canbay et al.^14^ measured tissue oxidative markers on postoperative day 5, whereas our study evaluated plasma MDA and blood GSH at two predefined time points, postoperative days 3 and 7.

Poyrazoglu et al.^25^ later examined hyperbaric oxygen (HBO) and HBO preconditioning in Sprague-Dawley rats with colonic anastomoses^25^. In that study, relaparotomy was performed on postoperative day 5, and the authors evaluated bursting pressure, hydroxyproline, tissue MDA, MPO, SOD, GPx, interleukin (IL)-6, IL-10, and tumor necrosis factor-alpha (TNF-α). HBO increased bursting pressure and hydroxyproline, reduced MDA and MPO, increased antioxidant enzyme activity, and decreased inflammatory cytokine levels. The direction of the mechanical and lipid peroxidation findings is similar to our curcumin model. The main difference is that HBO appeared to produce a broader response involving collagen content, antioxidant enzymes, neutrophil-related oxidative activity, and inflammatory cytokines, whereas curcumin produced a more selective pattern: stronger anastomoses and lower plasma MDA without a clear increase in hydroxyproline or blood GSH. This supports the idea that different interventions may improve anastomotic strength through partially distinct biological pathways.

Raptis et al.^11^, in their long-term experimental experience with colonic anastomotic healing, emphasized that anastomotic repair is a multifactorial process and should not be interpreted through a single endpoint. Their review of experimental models highlighted the recurrent use of bursting pressure, hydroxyproline, inflammatory infiltration, neoangiogenesis, fibroblast activity, collagen deposition, and leakage rates to evaluate anastomotic integrity. This framework fits the pattern observed in the present study. The mechanical outcome was improved by curcumin, but the biochemical and histopathological variables did not all move in the same direction. Rather than weakening the mechanical result, this discrepancy suggests that total hydroxyproline concentration and conventional ordinal histological scores may be insufficient to fully explain the biomechanical quality of early anastomotic repair.

The findings can also be placed within the broader context of pharmacological enhancement of colonic anastomotic healing. In their meta-analysis, Øines et al.^26^ reviewed 56 therapeutic agents across 75 experimental studies and noted that several interventions, including iloprost, tacrolimus, erythropoietin, growth hormone, insulin-like growth factor-1, HBO, and matrix metalloproteinase inhibitors reproducibly improved anastomotic stability in preclinical models. They also emphasized that experimental research in this field frequently relies on surrogate mechanical endpoints such as bursting pressure and breaking strength, because spontaneous anastomotic leakage is uncommon in animal models. Our findings fit this preclinical framework. Curcumin appears to be a biologically plausible pharmacological candidate because it improved the main mechanical endpoint and reduced lipid peroxidation. However, as with many interventions in this field, the results should be interpreted as mechanistic and hypothesis-generating rather than as direct evidence for clinical prevention of anastomotic leakage.

Directly comparable studies evaluating the effect of curcumin on the histological healing pattern of colonic anastomoses are limited. Therefore, the histopathological findings of the present study should be interpreted indirectly in relation to curcumin-based gastrointestinal and surgical wound-healing models rather than as a direct replication of previous anastomotic studies. Santos et al.^20^ evaluated perioperative oral curcumin supplementation in Wistar rats undergoing abdominal surgery and reported lower overall inflammatory cell infiltration and blood vessel count in the curcumin group. They also observed lower preoperative C-reactive protein (CRP) levels, although postoperative CRP did not differ significantly between groups. However, their histological analysis was based on hematoxylin and eosin (H&E) sections and did not further characterize inflammatory cell subtypes or collagen composition. In the present study, curcumin did not produce a similarly consistent histopathological pattern in the colonic anastomosis model. PNL infiltration, MNC infiltration, and blood vessel neodevelopment were mainly associated with postoperative assessment day rather than curcumin treatment. This difference may reflect the distinct biological setting of a sutured colonic anastomosis, in which mechanical integrity, local bacterial load, ischemia, collagen remodeling, and time-dependent inflammation interact more complexly than in a general abdominal wound model.

Zhang and Zhang^27^ investigated a curcumin-loaded alginate nano-micelle designed to improve delivery to the colon and overcome the hydrophobicity-related limitations of native curcumin. Their study was not an anastomotic model, but it directly addressed colonic wound healing. The nano-micelle formulation showed favorable gastrointestinal release characteristics, antibacterial activity against common wound-related bacteria, and no relevant cytotoxicity at the tested concentration. In the rat colonic wound model, repaired tissue was evaluated by colonoscopy and H&E staining, and the authors reported increased protein and collagen concentration, increased transforming growth factor-beta 1 (TGF-β1) expression, and reduced nuclear factor kappa B (NF-κB) expression.

These findings are biologically compatible with curcumin-enhanced colonic repair, but they also highlight an important difference from the present study. In our model, standard curcumin administration improved mechanical strength without a significant increase in hydroxyproline. The more evident collagen-related response in Zhang and Zhang’s study^27^ may be related to the nano-micelle delivery system, the non-anastomotic wound model, different timing of tissue evaluation, or the use of molecular markers rather than total hydroxyproline alone. This comparison suggests that formulation and local bioavailability may be important determinants of curcumin’s measurable effects on collagen-related repair.

Hacim et al.^28^ evaluated curcumin in a terminal ileal defect model repaired with a parietal peritoneal patch. Curcumin was administered by gavage and was associated with more frequent neomucosa formation, higher serum antioxidant activity, increased SOD and GPx activity, and lower MDA and 8-hydroxy-2’-deoxyguanosine (8-OHdG) levels. These findings support the antioxidant rationale of curcumin in intestinal repair and are consistent with the lower plasma MDA levels observed in our study. However, their model assessed mucosal regeneration in the ileum, whereas our study evaluated the mechanical integrity of a sutured colonic anastomosis. Moreover, unlike their enzymatic antioxidant response, blood GSH did not show a clear treatment-related increase in our model, suggesting that curcumin-related antioxidant effects may vary according to tissue type, sampling compartment, and timing.

Yıldız et al.^29^ reported lower inflammation, collagenization, and fibrosis scores in the curcumin group than in the steroid and control groups in a 28-day rat tracheal injury model. However, although the distribution of histological scores appeared more favorable with curcumin than with corticosteroid treatment, the study mainly presented overall intergroup *p*-values and did not report separate pairwise curcumin-versus-steroid comparisons for each histological parameter. Therefore, their findings support a potential anti-inflammatory and antifibrotic effect of curcumin, but they should not be interpreted as definitive evidence that curcumin is superior to corticosteroids.

Beyond these model-specific comparisons, the wound-healing literature provides a biological framework for interpreting the present findings. Curcumin has been reported to influence several overlapping components of tissue repair, including suppression of excessive inflammatory signaling, reduction of oxidative injury, promotion of fibroblast migration, enhancement of collagen deposition and matrix remodeling, and modulation of epithelial regeneration^15-19^. In diabetic and impaired wound models, curcumin has also been associated with lower pro-inflammatory cytokine activity, improved antioxidant enzyme responses, and more organized granulation tissue formation^17^,^18^,^30^. Its effects on neovascularization appear to be model- and phase-dependent.

Some studies have shown that curcumin may support angiogenic activity and increase factors such as VEGF and TGF-β during wound repair, whereas other models suggest that curcumin may reduce excessive vascular and inflammatory responses when these processes are already amplified^20^,^31^. This dual behavior may help explain why the present study demonstrated a clear mechanical benefit and lower lipid peroxidation without a uniform increase in hydroxyproline, blood vessel neodevelopment, or inflammatory histological scores. Taken together, the available evidence suggests that curcumin should not be interpreted simply as a collagen-increasing agent. Rather, its contribution to anastomotic repair may involve modulation of the healing microenvironment, with effects on oxidative stress, inflammation, tissue remodeling, and the quality rather than only the quantity of newly formed repair tissue. This interpretation is consistent with our main finding that curcumin strengthened the colonic anastomosis despite incomplete parallel changes in conventional biochemical and histopathological markers.

### Limitations

This study has several limitations. First, although the rat colonic anastomosis model provides a controlled experimental setting, it cannot fully reproduce the multifactorial nature of human colorectal anastomotic leakage. The findings should therefore be interpreted as preclinical and mechanistic rather than directly applicable to clinical practice.

Second, anastomotic bursting pressure was used as the primary mechanical endpoint. Although this is a widely used parameter in early anastomotic healing, it reflects only one aspect of anastomotic integrity. Other biomechanical properties, such as tensile strength, elasticity, and resistance to delayed leakage, were not evaluated.

Third, the biochemical analysis was limited to total hydroxyproline concentration, plasma MDA, and blood GSH. Although hydroxyproline provides a quantitative estimate of total collagen content, it does not provide information about collagen type, maturation, organization, cross-linking, degradation, or extracellular matrix remodeling. More specific assessments of collagen metabolism, including collagen type I and III expression, matrix metalloproteinase activity, or gene expression analyses, were not performed. Similarly, systemic oxidative stress markers may not fully reflect local oxidative events within the anastomotic tissue.

Fourth, histopathological evaluation was based on conventional ordinal scoring of inflammatory cell infiltration, blood vessel neodevelopment, and fibroblastic activity. Although these parameters provided descriptive information about the cellular healing response, they did not allow detailed quantification of collagen density, collagen organization, epithelial regeneration, angiogenic signaling, or molecular inflammatory pathways. More detailed immunohistochemical or molecular markers, such as VEGF, TGF-β, Ki-67, matrix metalloproteinases, endothelial markers, or collagen subtypes, were not assessed. This may have limited the ability to explain the marked biomechanical effect of curcumin at the tissue level.

Finally, the study included only adult male Wistar Albino rats, a relatively small number of animals per group, two postoperative assessment points, and a single curcumin dose. Detailed information on randomization, blinding, curcumin formulation, vehicle, purity, and bioavailability was not available in the original experimental record. Further studies with larger samples, dose-response designs, longer follow-up, high-risk anastomosis models, and tissue-level biochemical, immunohistochemical, and molecular analyses are needed to clarify the effects of curcumin on extracellular matrix remodeling and colonic anastomotic healing.

## Conclusion

Curcumin administration improved the biomechanical stability of colonic anastomoses in rats, as demonstrated by increased anastomotic bursting pressure during the early healing period. This effect was accompanied by lower plasma MDA levels, suggesting reduced lipid peroxidation. However, curcumin did not produce a significant parallel increase in tissue hydroxyproline concentration or blood GSH levels, and the histopathological findings did not show a uniform treatment-related healing pattern.

These findings suggest that curcumin may enhance early colonic anastomotic strength through mechanisms related, at least in part, to modulation of oxidative stress rather than a simple increase in total collagen content. Further experimental studies are needed to clarify the tissue-level mechanisms, optimal formulation, dose-response relationship, and translational relevance of curcumin in colonic anastomotic healing.

## Data Availability

The data supporting the findings of this study are available from the corresponding author upon reasonable request.
